# Magnitude and Associated Factors of Unintended Pregnancy among Pregnant Women at Saesie Tsaeda Emba Woreda Eastern Zone of Tigray, North Ethiopia, 2018

**DOI:** 10.1155/2019/1694808

**Published:** 2019-12-24

**Authors:** Gebrehiwot Gebremariam Weldearegawi, Kidanemaryam Berhe Tekola, Berhane Fseha Teklehaymanot

**Affiliations:** ^1^Adigrat University College of Medicine and Health Science, Department of Public Health, Ethiopia; ^2^Department of Nutrition and Dietetics, School of Public Health, College of Health Sciences, Mekelle University, Mekelle city, Tigray, Ethiopia

## Abstract

**Background:**

Each year there were about 80 million women who experienced unintended pregnancy in the globe. In Ethiopia, around one third of women have experiences of unintended pregnancy. However, the magnitude of unintended pregnancy was not determined in the study area. Hence the aim of the study was to assess the magnitude and associated factors of unintended pregnancy among pregnant women.

**Methods:**

Institutional based cross-sectional study design was employed among 345 participants. Participants were selected by systematic random sampling. Data was collected though face to face interview by structured questioner. It was entered, clean and analyzed using SPSS version 20. Descriptive analysis was done to see the frequency, percentage, mean and standard deviation. Adjusted odds ratio was computed at 95% confidence level to determine the effect of independent variable on the outcome variable. Variable at *p* value < 0.05 was declared as statistically significant variable. Model goodness of fit was checked using Hosmer lemeshow test.

**Result:**

The overall magnitude of unintended pregnancy was 24.9%. Employed women were 60% less likely having unintended pregnancy (AOR 0.4, 95% CI: 0.015, 0.785).Single women were 1.4 times more likely reported unintended pregnancy (AOR 1.4, 95% CI: 1.005, 3.675). Unintended pregnancy among ever visited by health extension workers was 1.7 times higher than not visited (AOR 1.7, 95% CI: 1.09, 5. 128). Unintended pregnancy among who had information about family planning were about 70% less likely reported unintended pregnancy than their counterparties (AOR 0.3, 95% CI: 0 .067, 0.845). Marital status, occupational status, visited by health extension workers, having information about family planning, discussing with their partners about contraceptive were found the major factors of unintended pregnancy. Thus the district health office, Tigray regional health office and other stakeholder should work to improve family planning accessibility, awareness, and utilization to overcome the problem.

## 1. Introduction

Unintended pregnancy is a pregnancy either mistimed or unwanted which causes a major problem in developed and developing countries affects women, child, family and the society as a whole. It is also an important public health problem predisposes women to in maternal death, abortion, low birth weight, preterm birth and infant mortality, unsafe abortions and poor maternity care [1, 2]. In African including Ethiopia, the most common reasons for unintended pregnancy was mainly associated with contraceptive failure, not using modern family planning, women being single in marital status, having long distance to the nearest health facility, having five and more number of pregnancies, partner disapproval to use modern family planning and partner poor awareness on modern family planning [3–5].

Each year, an estimated of 80 million pregnant women experiencing unintended pregnancy worldwide, that was close to one-third were in the third-world countries with the highest rate among the Eastern, and Middle African countries, causes both demographic trends and people's health and well-being problems [6]. Despite different international declarations were passed to alleviate the problem, about 86 unintended pregnancies occurred for every 1000 women in Sub Saharan African countries each year, and one third of them tend to end with unsafe abortion in the same region [2].

Women with their pregnancies were not intended have been found associated with different maternal and new born problems health problems. Some of the common health problems were unsafe abortion, low birth weight, pre term birth, high maternal and child under weight [7].

As a strategy family planning is the most effective ways in reducing maternal mortality due to unwanted pregnancy, but Ethiopia is a country with a high rate of unmet need for family planning (22%) and highest maternal death (420 deaths/100,000 live birth) [8]. However, to alleviate the problem, the Ethiopian government had prepared a national reproductive health strategy that gives stress on reducing unintended pregnancy by improving contraceptive utilization rate (66%) [9], but the current modern family planning utilization rate was very low (35%) [8].

Different studies in Ethiopia were found that about one third of pregnant women were reported their recent pregnancies were unintended (Gebreamlak, 26%, Getu, 24.5%, Tosheme, 36.5%, Hinsermu 32%, Weldegebriel, 28%, and Wado YD, 35% ) [3, 4, 10–13], but the studies done in different part of the country were reported inconsistent results, and there was lacks of information on the determinants of unintended pregnancies. In addition the magnitude of unintended pregnancy in eastern Zone of Tigray regional state, Northen Ethiopia (The study area) was not yet determined. There for this study tried to assess the magnitude of unintended pregnancy and its associated factors pregnant women at Saesie tsaed emba district health facilities among pregnant women attending antenatal care.

## 2. Methods

### 2.1. Study Area and Period

The study was conducted at SaesieTsada emba district, found in eastern zone of Tigray; North Ethiopia. It is found at 887 km far from Addis Ababa the capital city of the country and 97 km from Mekele, the capital city of the Tigray regional state. The district consists 30 “kebeles” which is the smallest administrative unit according to Federal Republic of Ethiopia. The district has one primary hospital, seven health centers and 28 health posts to serve the total inhabitants of the district. The survey was conducted from February to May 2018.

### 2.2. Study Design

Institution based cross-sectional study was employed. All pregnant women who were attending ANC in the health facilities of saesie tseada- emba were considered as study population and all pregnant women who were attending the antenatal care in the selected health facilities who selected to give information were considered as sample study.

### 2.3. Sampling Size and Techniques

The required sample size was determined using single population proportion formula by taking 31% proportion of unintended pregnancies from similar study done at Bahirdar, Ethiopia [3] 95% confident interval, 5% margin of error and 5% none response rate assumptions. Finally 345 participants selected by systematic random sampling were recruited from randomly selected three health facilities (one primary hospital and two health centers). Participants in each health facilities were distributed proportionally to population size based on their antenatal care client flow. Women were excluded if they were too sick to participate or suffering from mental illness during the data collection time. Data was collected though face to face interview using pre tested structured questionnaires. Questionaries were modified after pretest was done according to the findings. The questioners were including socio demographic and economic characteristics related variables, reproductive history related variables and magnitude of unintended pregnancy related variables. Four data collators who speak local language (Tigrigna) and whose profession was midwifery degree holders was recruited as data collectors and three masters holder Public health professionals who speak local language were recruited as supervisors. Two days training was given for data collectors and supervisors by principal investigators.

Daily checkup for complete filling of the questionnaires was also conducted by supervisors and principal investigators.

### 2.4. Data Analysis

The preliminary data was coded, entered and cleaned by EPI data version 3.1 then transferred to SPSS version 20 for analysis. First descriptive statistics was done. Frequency, percentage and mean of unintended pregnancy were computed using SPSS version 20. Bivariate and multivariate analysis was done to determine the effect of independent variables on the outcome variable. Variables with *P* value <0.2 in the bivariate analysis model were transferred into a multivariate logistic regression model to adjust the cofounders. Finally statistical significance was declared at *P* value <0.05 in the final model. The multi-collinearity will be checked by using variance inflation factors (VIF) cutoff point of 10. Finally, the data will be presented with texts, tables and graphs.

## 3. Result

### 3.1. Part I: Socio Demographic and Economic Characteristics of Participants

A total of 345 currently pregnant women attending antenatal care services were included in study making a response rate of 100%. The largest proportion, 117(33.9%) were in the age group between 20 and 24 years. The mean age of participants was 25 with of SD ± 4.6. Majority, 317 (91.9%) were married. Concerning the ethnicity, 336 (97.4%) were belongs to Tigray Ethnic group, and again 110 (31.9%) were completed primary and secondary school. Regarding age at first marriage, 245 (71.0%) got married at the age of between 18 and 24 years, however, 76 (22.0%) were married at <18 years old (Tables 1–3).

### 3.2. Part II: Reproductive History and Magnitude of Unintended Pregnancies of Participants

The overall magnitude of unintended pregnancy of the study was 24.9% (20.3–29.6%) of which, 25.9% unwanted and 74.4% mistimed pregnancies. The most common reason mentioned by participants was due to not using modern family planning (41.7%). Significant proportion of participants (25.8%) had history of abortion; around 25% of them had two and more episodes abortions. On their information about family planning, 326 (94.5%) were ever heard about modern contraceptive methods, but only 129 (37.4%) were ever used any types of modern family planning. However, 21.7% of them used traditional methods. Participants were also asked if they know the advantage of contraceptive. Hence 202 (58.6%) replied contraceptive can prevented from unwanted pregnancy and 123 (35.7) replied contraceptive prevents from mistimed pregnancy.

### 3.3. Part III: Factors Associated with Unintended Pregnancy of Pregnant Women Attending Antenatal Care at S/T/Emba District, Tigray, Ethiopia, 2018

On this study, bivariate analysis model was computed. Accordingly maternal age, maternal occupational status, maternal marital status, ever visited by health extension workers for reproductive health services, monthly income, partner occupational status, number of children they had, ever heard about family planning and discussed about family planning with their partners were significant factors to unintended pregnancy. After adjusted all the confounders, Occupational status, marital status, ever visited by health extension workers for reproductive health services, had information on family planning and ever discussed about family planning with their partners were found significant associations variables with the outcome variable.

Employed pregnant women were 60% less likely having an experiences of unintended pregnancy as compared with those who were farmers in occupational status (AOR = 0.4, 95% CI: 0.015–0.785). Single women were 1.4 times more likely reported unintended pregnancy as compared to married women (AOR = 1.4, 95% CI: 1.005–3.675). Women who were not ever visited by health extension workers for reproductive services were 1.7 times more likely having unintended pregnancy than those who did ever visited (AOR = 1.7, 95% CI: 1.09–5.128). Unintended pregnancy among women who had information about family planning were 70% less likely than their counterparties (AOR = 0.3, 95% CI: 0.067–0.845). In addition, unintended pregnancy among women who did not ever discussed about family planning with their partners were 1.2 times higher than those who ever discussed (AOR = 1.2, 95% CI: 1.034–3.786).

## 4. Discussion

Ethiopia is a country with a high rate of unmet need for family planning was observed (22%), Again the country did not achieved the millennium development goal remaining with the highest maternal death (420 deaths/100,000 live birth) [8]. However, to alleviate the problem, the Ethiopian government had declared a national reproductive health strategy focused on reducing unintended pregnancy by improving contraceptive utilization rate (66%) [9], but the current modern family planning utilization rate was remain very low (35%) [8].

The overall magnitude of unintended pregnancy was found 24.9% (20.3–29.6%). The finding was lower than a study done Pakistan (38.2%) [14]. It was also lower than studies done in Ghana (70%) [5], Malawi (55.6%) [15], Democratic republic of Congo (51.4%) [6] and Nigeria (35.9%) [16]. The variations might be explained due to study period and study area differences. The other Possible reason might be in due to Ethiopia there are health extension workers who are assigned at community (in the health post) that services for an average of 5000 inhabitants and these professionals are giving health services though home visiting, hence the awareness on intended pregnancy might be better from the others African countries. In addition there might be difference in health coverage with in different areas of African countries.

The magnitude of the our study was also slightly lower than studies done in Dilla University referral hospital of Ethiopia (36.9%) [17], West Wollega, Ethiopia (36%) [10] and Hawassa town, Ethiopia (34%) [18]. The variations might be due to the study period and study areas differences. As time goes up the awareness and utilization of family planning is expected to be raised, which intern helps to have wanted and planned pregnancies. In addition there might be difference in health coverage with in different areas of Ethiopia. However, the study shared similar findings with studies done in Felege Hiwot Referal hospital, Ethiopia, Mekelle city, North Ethiopia, Israel and Gelemso General hospital of South east Ethiopia [3, 12, 19, 20].

The Unintended pregnancy among participants who did not ever discuss on family planning methods with their partners/spouse were found high than those who had ever discussed. Similar finding were observed in different studies done in Ethiopia (in West Wollega, Hawassa town, Arba minch, and West Bellessa Woreda [10, 18, 21, 22]. This is the fact that partner discussion on family planning might enhance women decision power on contraceptive utilization rate to have planned pregnancies.

Our study also show, unintended pregnancy was found high among who did not visited by HEWs. Similar studies done at Arsi Negelle oromia region of Ethiopia and Ghana also explained visiting by health care providers had significant contribution to decrease unintended pregnancy [23, 24]. It could be explained by the health extension workers/ health care providers implemented community based health education programs that raise community awareness on the advantage of family planning.

Participants who were single during the study period were 1.4 times more likely reported unintended pregnancy than married women. The same evidences was also reported from studies done in Different parts of Ethiopia (Gonda, Dilla University Hospital, Gelemso General Hospital and Arsi Neglle) [7, 17, 20, 23]. The scenario could be due to single women might have unplanned sexual intercourse that lead them for unintended pregnancies as far as they did not have a stable union.

In the other way, participants employment was found as a protective effect for unintended pregnancy, it is the fact that participants who were employed during the study was about 60% less likely risk for unintended pregnancy than those who were farmers in the occupational status. This revealed that employed participants might have an access of information regarding family planning in their work places and might have the chance to read different books concerning on the important of planned pregnancies. Again they can share information from colleagues.

Participants who ever had information about family planning were also 70% less likely experienced unintended pregnancy than their counterparties. This finding was in line with a studies done at Arbaminch town, Ethiopia [25] and in Democratic republic of Congo [6]. This might revealed that information towards family planning may be a clue for deciding the number of children they have in their lifespan and it makes their episodes of pregnancies might be intended. However, Participants age, Monthly income, husband educational status and number of children they have did not show any level of association with unintended pregnancy in the study area.

### 4.1. Conclusion and Recommendation

In our study, almost one third of the participants were found their recent pregnancy was unintended. Marital status, occupational status, visited by health extension workers, having information about family planning, discussing with their partners about contraceptive were found the factors that contribute for unintended pregnancies. Thus the district health office in collaborations with Tigray regional health bureau and other stakeholder should work to improve family planning accessibility, awareness, and utilization to overcome the problem.

### 4.2. Limitation of the Study

- As far as the study was cross sectional study it did not address the causality of unintended pregnancy
- The study was conducted in one district so the findings cannot be assumed to be the same in other settings.

## Figures and Tables

**Table 1:** Socio-demographic and economic characteristics of pregnant women attending anti natal care at saesie tsaed emba district eastern Tigray Northern Ethiopia, 2018 (*n* = 345).

Demographic variables	Characteristics	Frequency	Percentage
Age	15–19	16	4.6
	20–24	117	33.9
	25–29	89	25.8
	30–34	61	17.7
	35–39	52	15.5
	>40	10	2.9
	Total	345	100%

Ethnicity	Tigray	336	97.4
	Other	9	2.6
	Total	345	100.0

Religion	Orthodox	328	95.1
	Muslim	14	4.1
	Others	3	0.9
	Total	345	100.0

Education	Illiterate	97	28.1
	Grade 1–4	40	11.6
	Grade 5–8	59	17.1
	Grade 9–10	113	32.8
	Grade 11–12	6	1.7
	Diploma	16	4.6
	First degree	14	4.1
	Total	345	100.0

Occupational status	Farmer	52	15.1
	Student	9	2.6
	Government/private employee	96	28
	Merchant/private work	45	13
	House wife/unemployed	204	59.
	Total	345	100.0

Monthly income	<500	96	27.8
	501–1000	100	29.1
	1001–2000	71	20.6
	2001–3000	47	13.6
	>3001 birr	31	9.0
	Total	345	100.0

Marital status	Married	317	91.9
	Single	11	3.2
	Divorced	10	2.9
	Others (Widowed and separated)	7	2
	Total	345	100.0

Years of marriage	<18 years	76	22.0
	18–24 years	245	71.0
	25–30 years	13	4.5
	Total	334	97.4

Number of children have	1–2	140	40.6
	3–5	149	43.2
	>5	56	16.2
	Total	345	100.0

Husband educational status	Can't read and write	36	10.4
	Only read and write	65	18.8
	Primarily level	110	31.9
	Secondary level	65	18.8
	Above secondary level	40	11.6
	Total	316	91.6

Husband occupation	Farmer	141	40.9
	Private work	66	19.1
	Government employee	49	14.2
	Daily labor	21	6.1
	Merchant	29	8.4
	Unemployed/farmer	10	2.9
	Total	316	91.6

Do you have radio	Yes	250	72.5
	No	95	27.5
	Total	345	100.0

Do you have TV	Yes	130	37.7
	No	215	62.3
	Total	345	100.0

**Table 2:** Reproductive history and modern family planning utilization of pregnant women attending antenatal care at Saesie Tsaeda emba District health facilities, Eastern Zone of Tigray, North Ethiopia, 2018 (*n* = 345).

Variables	Characteristics	Frequency	Percentage
Number of pregnancy (*n* = 345)	One	130	37.7
	Two	67	19.4
	Three	41	11.9
	Four	43	12.5
	Five and above	64	18.6
	Total	345	100.0

Status of current pregnancy (*n* = 345)	Unintended	86	24.9
	Intended	259	75.1
	Total	345	100.0

Types of unintended (*n* = 86)	Unwanted	22	25.94
	Mistimed	64	74.41
	Total	60	100

Cause of unintended pregnancy (*n* = 86)	Not use of using modern family planning	35	40.7
	Contraceptive failure	15	17.4
	No awareness about FPMS	21	24.4
	Am not consider that I am pregnant	15	17.4
	Total	86	100

Previous experience of unintended pregnancy	Yes	27	7.8
	No	318	92.2
	Total	345	100.0

Period of last unintended pregnancy occurred	Within the last three years	10	37.03
	After 3 years	17	62.96
	Total	27	100

Number of children you have (*n* = 354)	Only 1	187	54.2
	Two and above	127	36.8
	None	31	9
	Total	345	100

Do you have history of abortion (*n* = 345)	Yes	69	20
	No	276	80
	Total	345	100.0

Number of history of abortion (*n* = 69)	One	51	73.9
	Two and above	18	25.1
	Total	69	100

Have you ever visited by HEW reproductive health services (*n* = 345)	Yes	291	84.3
	No	54	15.7
	Total	345	100.0

Have you an information about FP services (*n* = 345)	Yes	326	94.5
	No	19	5.5
	Total	345	100.0

Source of information for family planning services (*n* = 345)	Radio	18	5.2
	Tv	22	6.4
	Friends	21	6.1
	HEW	250	72.5
	Health care provider	15	4.3
	Missing System	19	5.5
	Total	345	100.0

Have you ever used modern family planning methods? (*n* = 345)	Yes	129	37.4
	No	216	62.6
	Total	345	100.0

Which modern family planning did you heard? (*n* = 129)	Pills	83	24.1
	Injectable	145	41.6
	IUCD	18	5.2
	Norplant	59	17.1
	Condom	20	5.8
	Female sterilization	1	0.3
	Total	291	100.0

Reasons not ever used modern family planning methods (*n* = 216)	Not aware of MCM	24	18.6
	Use traditional methods	28	21.7
	Unacceptable in my culture	21	16.3
	Fear of side effects	36	27.9
	Husband disapproval	20	15.5
	Total	129	100.0

Do you know the advantages of the modern contraceptive methods (*n* = 345)	To prevent unwanted pregnancy	202	58.6
	To delay mistimed pregnancy	123	35.7
	I do not know	20	5.8
	Total	345	100.0

Who decides to use contraceptive before your current pregnancy (*n* = 345)	My self only	53	15.4
	My husband only	33	9.6
	Myself and my husband jointly	253	73.3
	Other	6	1.7
	Total	345	100.0

**Table 3:** Factors associated with unintended pregnancy for pregnant women attending antenatal care at SaesieTsa edaemba District, Eastern Zone of Tigray, North Ethiopia, 2018 (*n* = 345).

S.N	Variable	Characteristics	Un intended pregnancy		*COR*	AOR
			Yes	No		
1	Age of mothers	15–19 yrs	6 (8.8%)	11 (3.9%)	1.4 (0.284–0.472)	.1 (0.002–7.503)
		20–24 yrs	12 (21.2)	104 (36.5)	**5.3 (1.339–1.734)**	1.1 (.032–8.877)
		25–29 yrs	17 (28.3)	72 (25.3)	2.8 (0.716–11.124)	.9 (.032–3.317)
		30–34 yrs	15 (25)	46 (16.1)	2.04 (0.716–8.23)	11.7 (.388–15.051)
		35–39 yrs	6 (10)	46 (16.1)	**5.1 (1.113–23.47)**	0.5 (0.223–18.243)
		>=40 yrs	4 (6.7)	692,1	1	1

2	Occupation	Farmer	9 (5)	43 (15.1)	1	1
		Student	5 (8.3)	4 (1.4)	0.83 (0.279–8.596)	0.7 (.546–5.342)
		Gov't/private employed	7 (11.7)	28 (9.8)	**0.16 (0.034–0.259)**	.4 (0.015–0.785)^∗^
		Merchant/private work	5 (8.5)	40 (14)	1.67 (0.413–1.893)	1.2 (0.674–13.654)
		House wife	34 (56.7)	170 (59.7)	1.04 (0.108–2.777)	1.03 (0.764–4.167)

3	Marital status	Married	48 (80)	269 (94.6)	1	1
		Single	5 (8.3)	6 (2.1)	**2.14 (1.063–7.382)**	**1.4 (1.005–3.675) ** ^∗^
		Divorced/separated	7 (11.7)	10 (3.6)	**0.12 (0.04–0.91)**	1.3 (0.491–3.283)

4	Ever visited by Hews for Rh services	Yes	44 (73.3)	247 (86.7)	**1**	1
		No	16 (26.7)	38 (13.3)	**2.3 (1.214–4.602)**	**1.7 (1.09–5.128) ** ^∗^

5	No of children	2 and above	41 (68.3)	242 (84.9)	**1**	1
		One or none	19 (31.7)	43 (15.1)	**2.6 (1.38–4.91)**	3 (0.987–6.892)

6	Monthly income	<500	17 (28.4%)	79 (27.7%)	**0.051 (0.005–0.547) ** ^∗^	0.49 (.136–1.829)
		501–1000	21 (35%)	43 (15.1%)	**0.062 (0.007–0.569) ** ^∗^	.53 (.143–1.919)
		1001–2000	12 (20%)	87 (30.5%)	**0.063 (0.008–0.510) ** ^∗^	.34 (.092–1.261)
		>2000	10 (16.6)	76 (26.7)	1	1

7	Have information about FP	Yes	54 (90)	272 (95.4)	**0.43 (0.187–0.786)**	**0.3 (.067–.845) ** ^∗^
		No	6 (10)	13 (4.6)	1	1

8	Ever discussed with partner about FP	Yes	33 (55)	199 (69.8)	1	1
		No	27 (45)	86 (30.2)	1.8 (1.073–3.341)	**1.2 (1.034–3.786) ** ^∗^

The bold value is indicated the variables associated factors with the out come variable.

## Data Availability

All data pertaining to this study are contained and presented in the document at the annex.

## Abbreviations

AOD: Adjusted odds ration
ANC: Anti natal care
CI: Confidence interval
HEWs: Health extension workers
NGOs: Nongovernmental organizations
SPSS: Statistical package for social science.
